# Supplementary material for: SH3RF2 contributes to cisplatin resistance in ovarian cancer cells by promoting RBPMS degradation
Source: Commun Biol. 2024 Jan 9;7:67. doi: 10.1038/s42003-023-05721-1 (PMC10776562; doi:10.1038/s42003-023-05721-1)
Supplement: Supplementary file 2 — Supplementary Information [file 42003_2023_5721_MOESM2_ESM.pdf]

Supplementary Information

SH3RF2 contributes to cisplatin resistance in ovarian cancer cells by promoting RBPMS degradation

Ting-Ting Gong [1 #], Fang-Hua Liu [2 #], Qian Xiao [1, 2 #], Yi-Zi Li [2], Yi-Fan Wei [2], He-Li Xu [2], Fan Cao [2], Ming-Li Sun [1], Feng-Li Jiang [1], Tao Tao [1], Qi-Peng Ma [1], Xue Qin [1*], Yang Song [1], Song Gao [1], Lang Wu [3], Yu-Hong Zhao [2, 4], Dong-Hui Huang [2, 4*], Qi-Jun Wu [1, 2, 4, 5*]

[1]Department of Obstetrics and Gynecology, Shengjing Hospital of China Medical University, Shenyang, China.

[2]Department of Clinical Epidemiology, Shengjing Hospital of China Medical University, Shenyang, China.

[3]Cancer Epidemiology Division, Population Sciences in the Pacific Program, University of Hawaii Cancer Center, University of Hawaii at Manoa, Honolulu, Hawaii, USA.

[4]Liaoning Key Laboratory of Precision Medical Research on Major Chronic Disease, Shengjing Hospital of China Medical University, Shenyang, China.

[5]NHC Key Laboratory of Advanced Reproductive Medicine and Fertility (China Medical University), National Health Commission, Shenyang, China.

[#]These authors contributed equally to this work.

Corresponding to:

Qi-Jun Wu, M.D., Ph.D.

Department of Obstetrics and Gynecology, Department of Clinical Epidemiology, Shengjing Hospital of China Medical University, Liaoning Key Laboratory of Precision Medical Research on Major Chronic Disease, Shengjing Hospital of China Medical University. NHC Key Laboratory of Advanced Reproductive Medicine and Fertility (China Medical University), National Health Commission.

Address: No. 36, San Hao Street, Shenyang, Liaoning 110004, P. R. China

Phone: 86-24-96615-13652

E-mail: wuqj@sj-hospital.org

AND

Dong-Hui Huang, M.D., Ph.D.

Department of Clinical Epidemiology, Shengjing Hospital of China Medical University. Liaoning Key Laboratory of Precision Medical Research on Major Chronic Disease, Shengjing Hospital of China Medical University.

Address: No. 36, San Hao Street, Shenyang, Liaoning 110004, P. R. China

Phone: 86-24-96615-13652

E-mail: huangdh_cc@163.com

AND

Xue Qin, M.D.

Department of Obstetrics and Gynecology, Shengjing Hospital of China Medical University.

Address: No. 36, San Hao Street, Shenyang, Liaoning 110004, P. R. China

Phone: 86-24-96615-41311

E-mail: qinx@sj-hospital.org

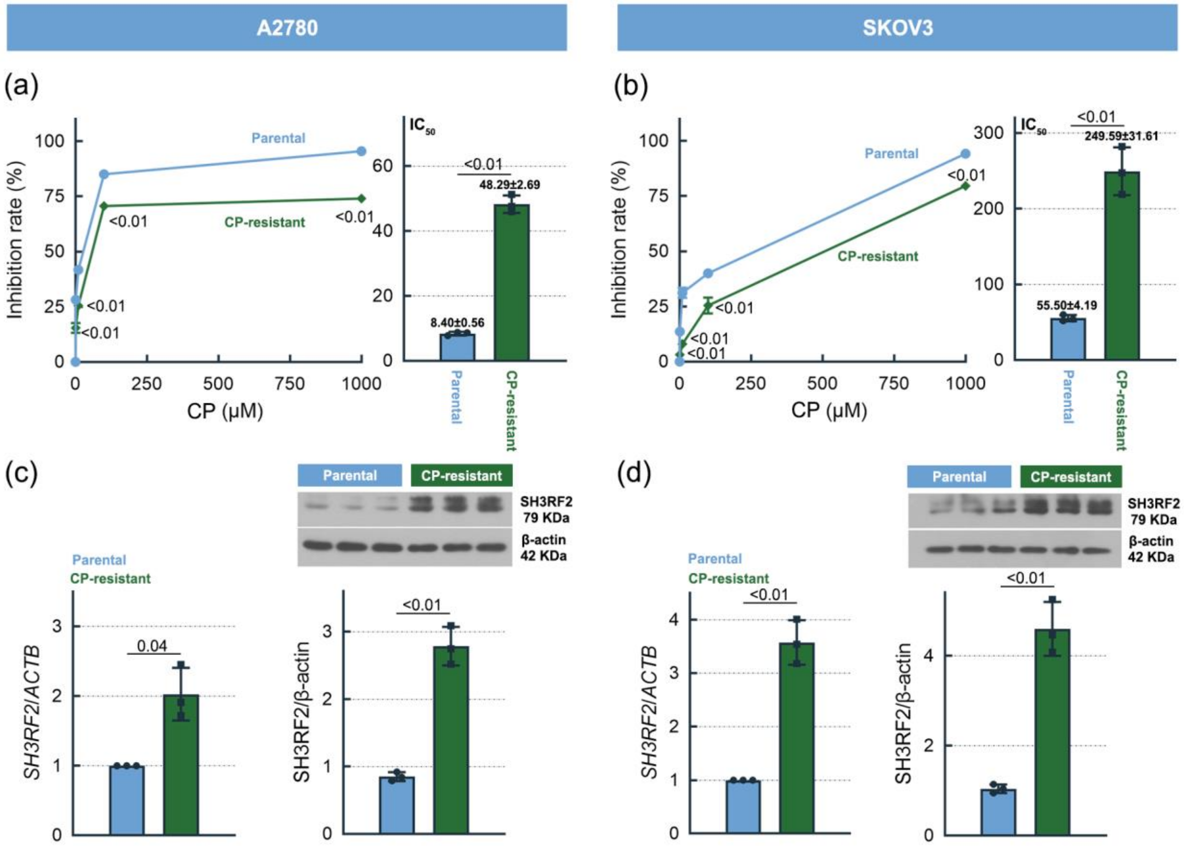

Supplementary Fig.1 Expression of SH3RF2 in CP-resistant OC cells.

(a, b) Parental and CP-resistant OC cells were exposed to 0, 1, 10, 100, or 1000 μM CP for 48 h and then MTT assays were performed ($p < 0.01$ vs. the parental group). n=3. The IC50 values of CP in parental and CP-resistant OC cells were calculated and the CP-resistant OC cells showed significantly increased IC50 values of CP. n=3. (c, d) The mRNA and protein expression of SH3RF2 in parental and CP-resistant OC cells were measured by RT-qPCR (n=3) and western blotting (n=3). Data are expressed as the mean ± SD. The p values were determined by two-way ANOVA, unpaired Student's t-test, or Welch's t test.

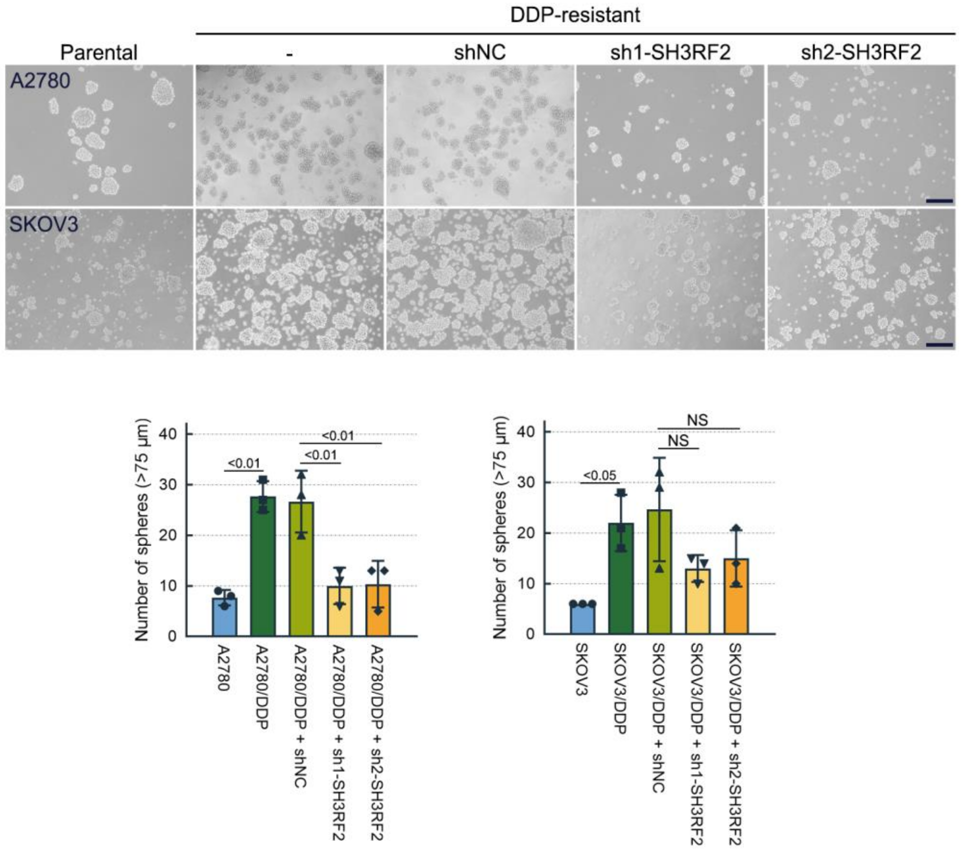

Supplementary Fig.2 Effect of SH3RF2 depletion on the stem-like properties of DDP-resistant OC cells.

Parental and DDP-resistant OC cells were used to perform sphere formation assays (Scale bars, 200 μm). After 10 days, the spheres with the diameter of >75 μm were counted. n=3. Data are expressed as the mean ± SD. The p values were determined by one-way ANOVA.

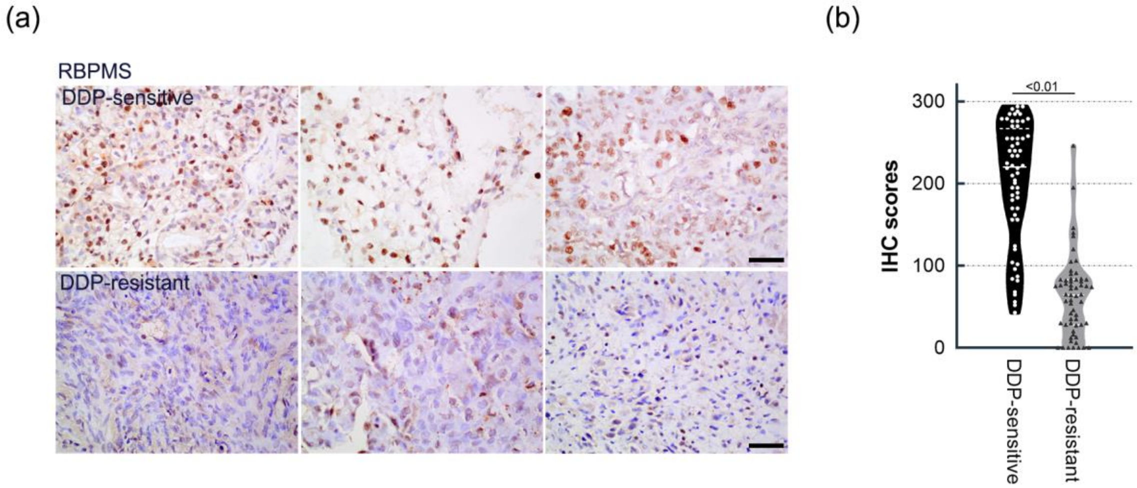

Supplementary Fig.3 Expression of RBPMS in the tumor tissues from DDP-sensitive and DDP-resistant OC patients.

(a) Representative images of IHC staining of RBPMS in the tumor tissues from DDP-sensitive and DDP-resistant OC patients (Scale bars, 50 μm). (b) The quantification of RBPMS staining was performed by two pathologists. n=60. The p values were determined by Mann-Whitney U test.

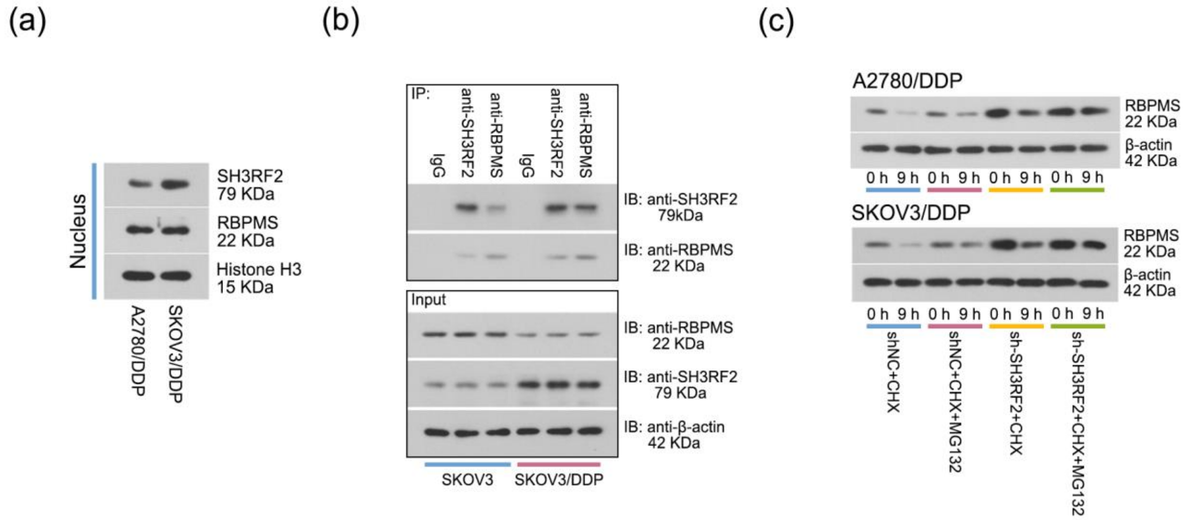

Supplementary Fig.4 Interaction between SH3RF2 and RBPMS.

(a) Expression of endogenous SH3RF2 and RBPMS in the nucleus of DDP-resistant A2780 and SKOV3 cells detected by western blotting. n=3. (b) Co-immunoprecipitation of SH3RF2 with RBPMS using anti-SH3RF2 and anti-RBPMS. Immunoblotting was performed with anti-RBPMS and anti-SH3RF2. n=3. (c) DDP-resistant A2780 and SKOV3 cells with stable depletion of SH3RF2 were treated with 20 μg/mL CHX or/and 5 μM MG132 for 0 and 9 h. Western blots of lysates from cells stained for RBPMS. n=3.

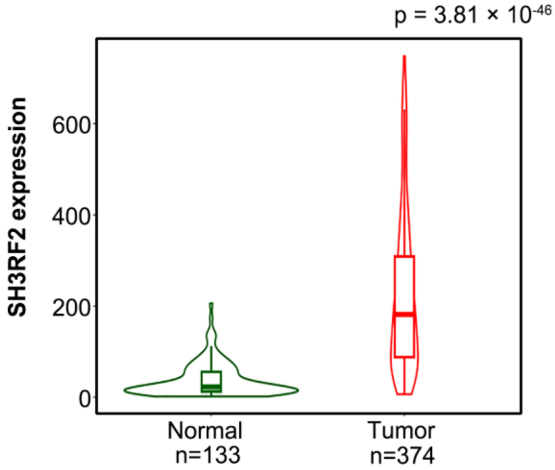

Supplementary Fig.5 SH3RF2 expression levels were higher in the OC tissues compared to the normal tissues.

Expression levels of SH3RF2 in the ovarian serous cystadenoma tissues (Tumor, n = 374) and the normal ovary tissues (Normal, n = 133) from the TNM plot database (https://tnmplot.com/analysis/). The p values were determined by Mann-Whitney U test.

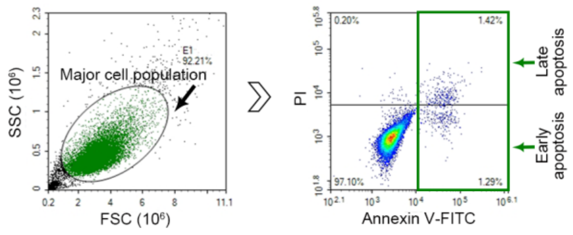

Supplementary Fig.6 Flow cytometry gating strategy for Annexin V-FITC/PI staining.

Fig.2d and 2e

WB: SH3RF2 WB: β-actin

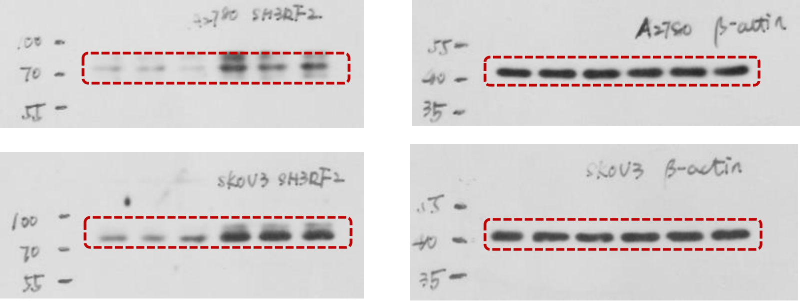

Fig.3a

WB: SH3RF2 WB: β-actin

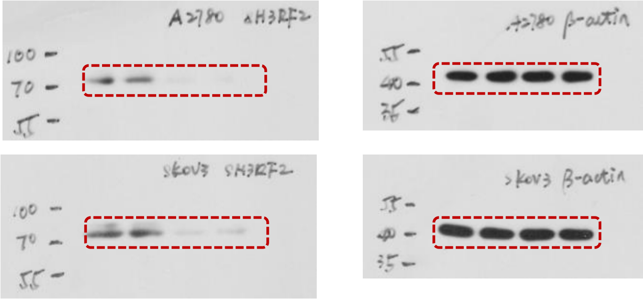

Fig.3e

WB: cleaved PARP

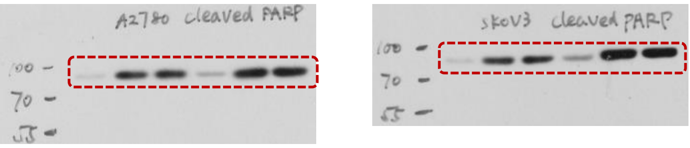

WB: cleaved caspase 3

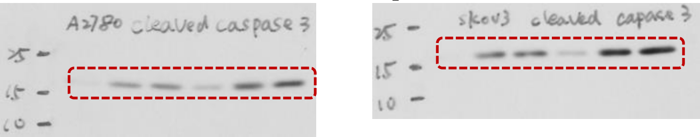

WB: β-actin

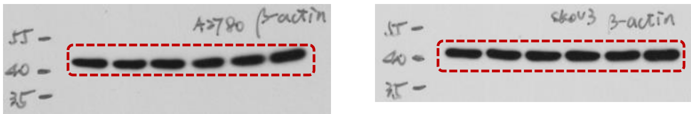

Fig.4c

WB: γH2AX

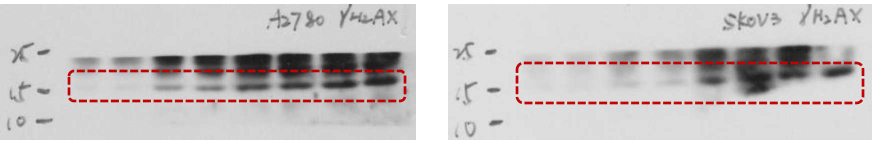

WB: β-actin

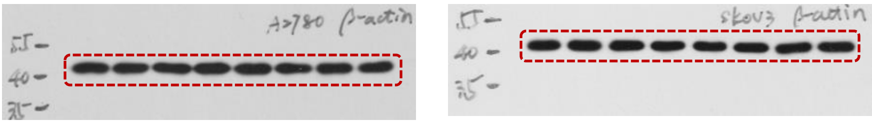

Fig.6e

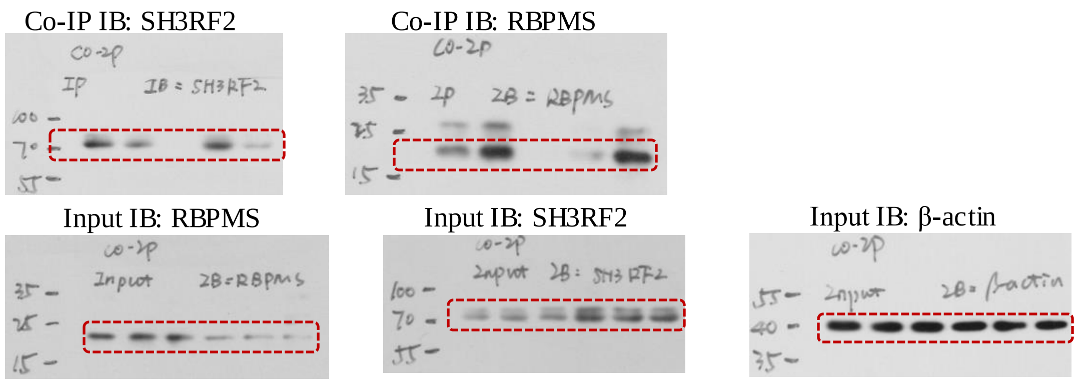

Fig.6f

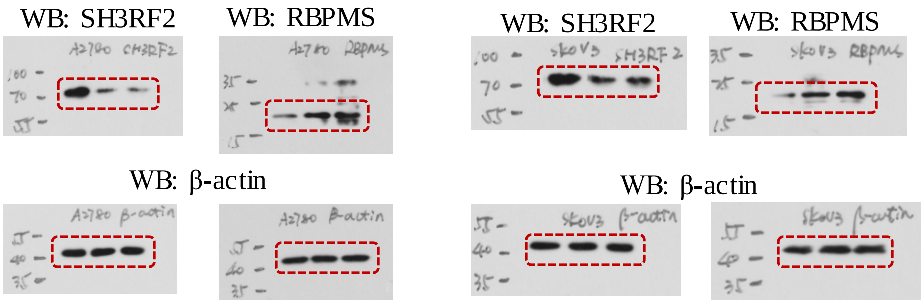

Fig.6g

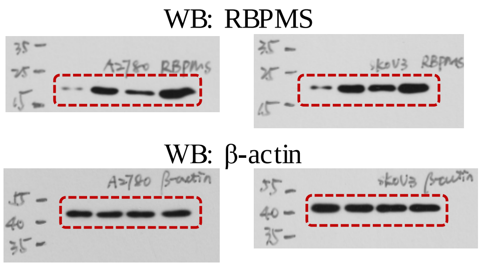

Fig.6h

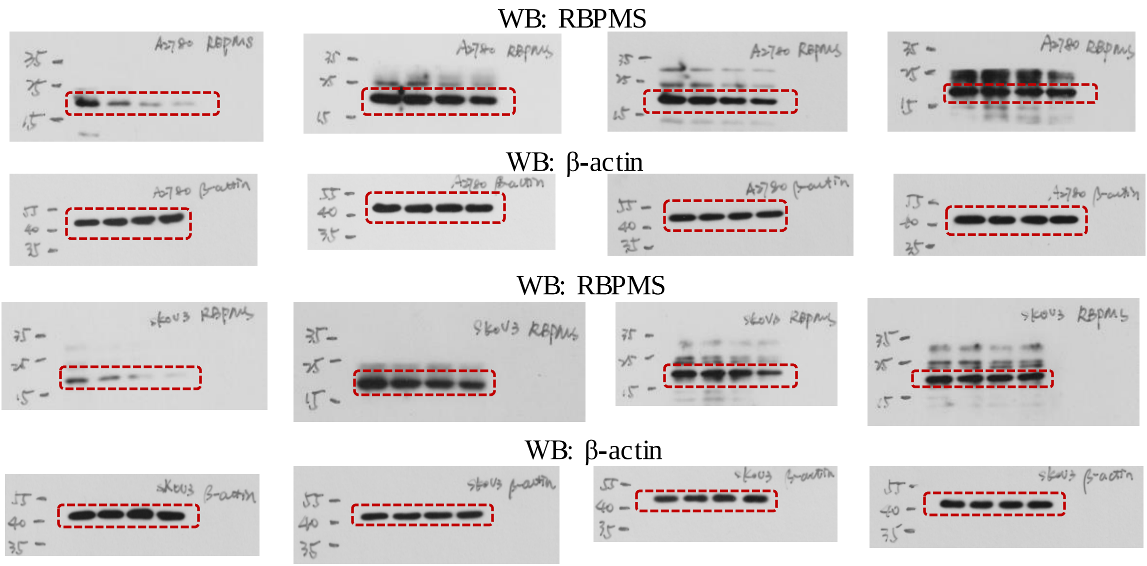

Fig.6i

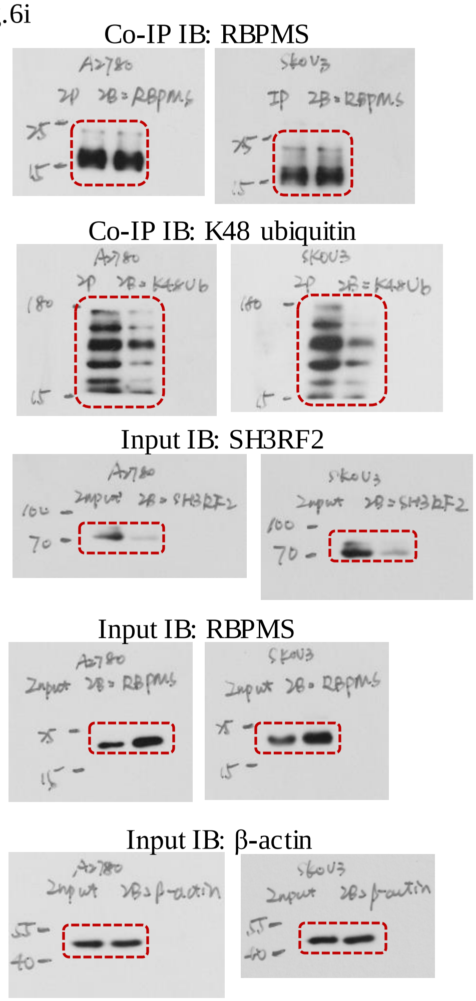

Fig.6j

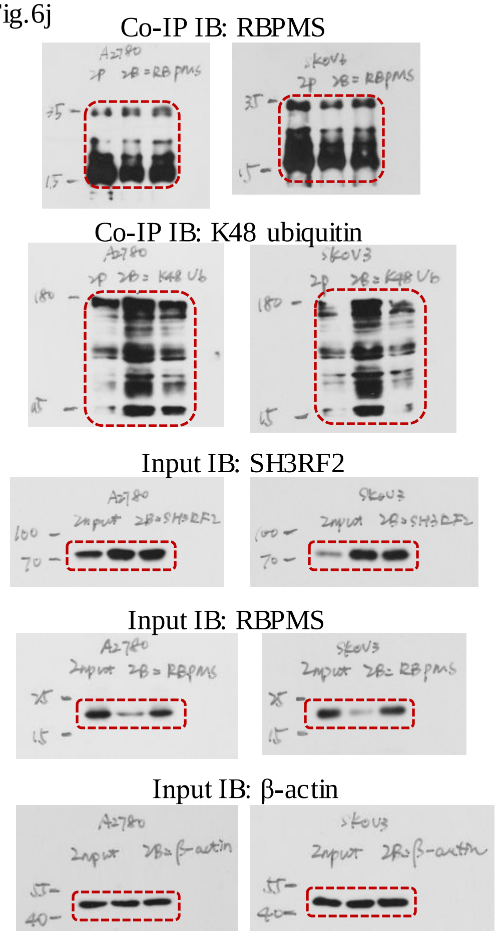

Fig.8a

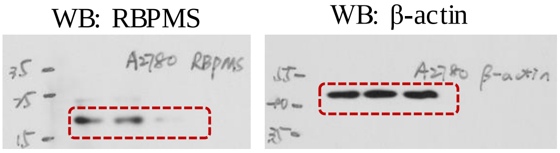

Fig.8b

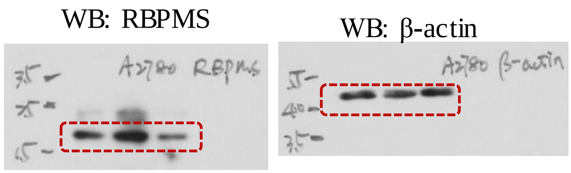

Supplementary Fig.1c and 1d

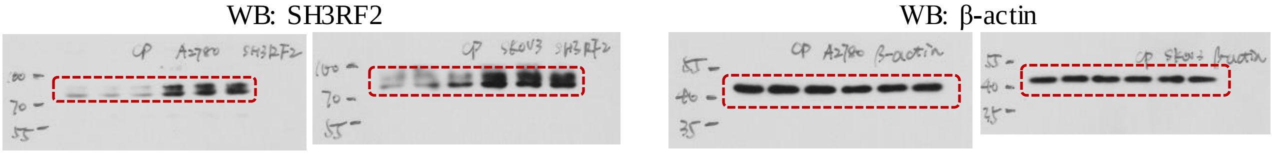

Supplementary Fig.4a

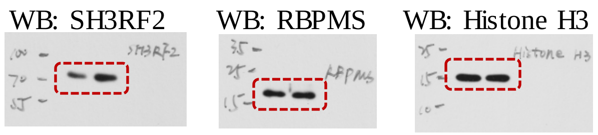

Supplementary Fig.4c

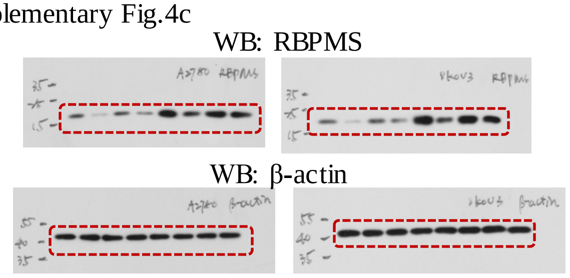

Supplementary Fig.4b

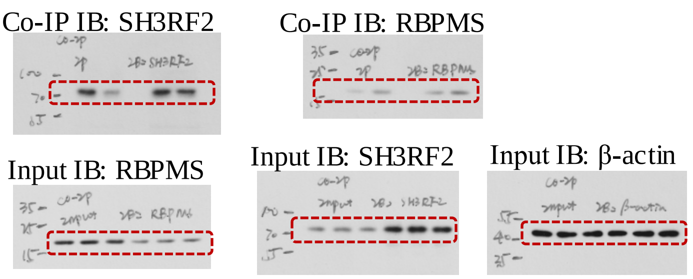

Supplementary Fig.7 Uncropped western blot images.
